# Cardioprotective action of aprepitant in a rat model of ischemia-reperfusioninduced myocardial injury: role of PI3K-AkT-GSK-3β-HIF-1α signaling pathway

**DOI:** 10.1590/acb371004

**Published:** 2022-12-19

**Authors:** Mei Qian, Yang Liu

**Affiliations:** 1 MM. Taizhou Second People’s Hospital – Department of Pharmacy – Taizhou, China.; 2 MM. Mudanjiang Medical University – Teaching Materials Section – Mudanjiang, China.

**Keywords:** Substance P, Receptors, Neurokinin-1, Isolated Heart Preparation, Ischemia, Rats

## Abstract

**Purpose:**

The present study explored the role and mechanism involved in aprepitant-induced cardioprotective effects in rat model of ischemia-reperfusion injury.

**Methods:**

The isolated hearts of Wistar male albino rats were subjected to ischemia-reperfusion injury on Langendorff apparatus. The extent of myocardial injury was assessed by measuring lactate dehydrogenase 1 and CK-MB release in the coronary effluent. The rats were treated with aprepitant (5, 10 and 20 mg/kg) before isolating hearts. After injury, the levels of HIF-1α, p-AkT, p-GSK-3β/GSK-3β were measured in heart homogenates. LY294002 was employed as PI3K inhibitor.

**Results:**

Ischemia-reperfusion led to significant myocardial injury and decreased the levels of HIF-1α, p-AkT and ratio of p-GSK-3β/GSK-3β. Aprepitant attenuated myocardial injury and restored the biochemical changes in a dose-dependent manner. Pre-treatment with LY294002 (10 and 20 mg/kg) abolished aprepitant-mediated cardioprotective effects and restored the biochemical parameters in the heart homogenate.

**Conclusions:**

Aprepitant may be effective in preventing ischemia-reperfusion-induced myocardial injury, which may be due to activation of PI3K-AkT-GSK-3β and HIF-1α signaling pathway.

## Introduction

Ischemia-reperfusion injury remains one of the major causes of cell death through the world, and ischemia-reperfusion-induced myocardial injury remains at the top of all diseases^1^. Despite a lot of research done in this area, there is still a paucity of good pharmacological agents that can be successfully employed to manage or prevent ischemia-reperfusion-induced myocardial injury. Therefore, there is a need to identify the novel agents to attenuate or ameliorate ischemia-reperfusion-induced myocardial injury.

Aprepitant acts as a specific neurokinin 1 (NK1-R) blocker^2^. It is officially approved for the treatment of chemotherapy-induced nausea and vomiting, and it produces this action by blocking the actions of substance P^3^^,^ 4. It is also reported to produce other beneficial actions like attenuating cisplatin-induced hepatotoxicity and nephrotoxicity^5^. It is also been projected as a very good anticancer drug^6^. Moreover, substance P has been shown to regulate basal cardiovascular functions in humans^7^. Substance P has been shown to increase oxidative stress by increasing the production of reactive oxygen species^8^. In turn, the production of free radicals is linked with heart injury and failure^9^. Administration of aprepitant has been shown to prevent viral-induced myocarditis in mice along with significant improvement in myocardial contractility^10^. Moreover, in cell line studies, the use of aprepitant has been shown to prevent doxorubicin-induced reduction of cell viability, apoptotic cell death, and oxidative stress in cardiomyocytes^11^. It has been shown that aprepitant attenuates doxorubicin-induced cardiac injury in mice in a significant manner^12^.

Based on these studies, it may be hypothesized that aprepitant may be useful in attenuating ischemia-reperfusion-induced heart injury. Therefore, the present study was designed to explore the role and mechanism involved in aprepitant-induced cardioprotective effects in rat model of ischemia-reperfusion-injury on the Langendorff system.

## Methods

Male Wistar albino rats (200-250 g) were taken for the study and were exposed to 12 hours of light and 12 hours of dark in standard conditions of animal house. The experimental protocol was approved by the Animal’s Ethical Committee of Mudanjiang Medical University and approval number: MDJ.No20211030b060. The doses of aprepitant^5^ and LY294002^13^^,^ 14 were selected on the basis of previous studies. The kits for the quantification of lactate dehydrogenase 1 (LDH-1), CK-MB isoform of creatine kinase, HIF-1α, p-AkT and p-GSK-3β were procured from MyBioSource, Inc. (San Diego, CA, United States of America).

### Ex-vivo model of ischemia-reperfusion injury on Langendorff apparatus

Animals were administered different doses of aprepitant (5, 10 and 20 mg/kg intraperitoneal route). After 30 minutes of injection, animals were sacrificed, and hearts were removed very quickly. The hearts were perfused on the Langendorff apparatus with Kreb’s Henseleit (KH) solution at 37 °C^15^. The inflow of KH solution was stopped for 30 minutes to induce ischemia to whole rat heart. Afterwards, KH flow was restored for 2 hours during which reperfusion injury was induced^16^^,^ 17.

### Myocardial injury parameters

The extent of heart injury was assessed by measuring the release of heart-specific biochemicals, i.e., LDH-1 and CK-MB in the coronary effluent using commercially available kits.

*Quantification determination of HIF-1*α*, p-AkT, and p-GSK-3*β *levels*

After reperfusing the hearts on Langendorff apparatus, the hearts were removed and homogenized in phosphate buffer saline solution. Within the heart homogenate supernatants, the levels of HIF-1α, p-AkT and p-GSK-3β/GSK-3β were measured using commercially available enzyme-linked immunosorbent assay (ELISA) assay kits.

### Experimental design

Seven experimental groups were used, and each group had eight animals:

- Non-ischemic group: hearts were not subjected to any ischemia or reperfusion, and these hearts were isolated for the biochemical estimations of HIF-1α, p-AkT, and p-GSK-3β/GSK-3β;
- I/R injury group: hearts were subjected to 30 minutes of ischemia and 120 minutes;
- Aprepitant (5 mg/kg) in ischemia-reperfusion (IR) injury: aprepitant (5 mg/kg) was administered to rats 30 minutes before isolating the hearts, and it was followed by 30 minutes of ischemia and 120 minutes of reperfusion;
- Aprepitant (10 mg/kg) in IR injury: Aprepitant (10 mg/kg) was administered to rats 30 minutes before isolating the hearts, and it was followed by 30 minutes of ischemia and 120 minutes of reperfusion;
- Aprepitant (20 mg/kg) in IR injury: Aprepitant (20 mg/kg) was administered to rats 30 minutes before isolating the hearts, and it was followed by 30 minutes of ischemia and 120 minutes of reperfusion;
- Aprepitant (20 mg/kg) and LY294002 (10 mg/kg) in IR injury: LY294002 (10 mg/kg) was given to rats 30 minutes before aprepitant (20 mg/kg), which in turn was given to rats 30 minutes before isolating the hearts. It was followed by 30 minutes of ischemia and 120 minutes of reperfusion;
- Aprepitant (20 mg/kg) and LY294002 (20 mg/kg) in IR injury: LY294002 (20 mg/kg) was given to rats 30 minutes before aprepitant (20 mg/kg), which in turn was given to rats 30 minutes before isolating the hearts. It was followed by 30 minutes of ischemia and 120 minutes of reperfusion.

## Statistics

The data were represented as mean ± standard deviation (SD). The results were also represented as median with interquartile range. The statistical analysis was done using GraphPad Prism 9.4.1. The Shapiro-Wilk’s test was performed to determine normality of data (normal distribution test), and data was found to pass normality test (p < 0.05). The data of LDH-1 and CK-MB were analysed using two-way analysis of variance (ANOVA) because of the influence of two factors, i.e., time (before ischemia and after reperfusion) and treatment were analysed on the release of these cardiac-specific biomarkers. The other data were analysed using one-way ANOVA. Tukey’s post hoc test was used in all parameters. The statistical significance was fixed at *p* < 0.05. Considering the confidence level of 95%, the margin of error as 5%, population proportion as 0.6%, and population size of 56, the sample size came out as eigth. Accordingly, eight animals were included in each group.

## Results

### Ischemia-reperfusion leads to myocardial injury and induces biochemical changes in heart

**Figure 1:**
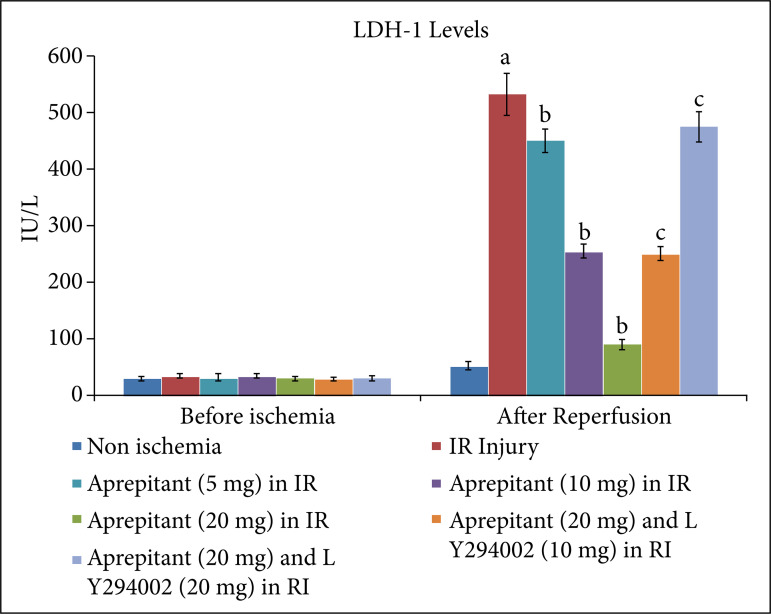
Effect of aprepitant and LY294002 on ischemia-reperfusion-induced LDH-1 release in the coronary effluent. Values are givenas mean ± SD. **(a)** *p* < 0.05 *vs*. non-ischemia; **(b)** *p* < 0.05*vs*. IR injury; **(c)** *p* < 0.05 *vs*. aprepitant (20 mg/kg).

LDH-1: lactate dehydrogenase 1; IR: ischemia-reperfusion; SD: standard deviation.

**Figure 2:**
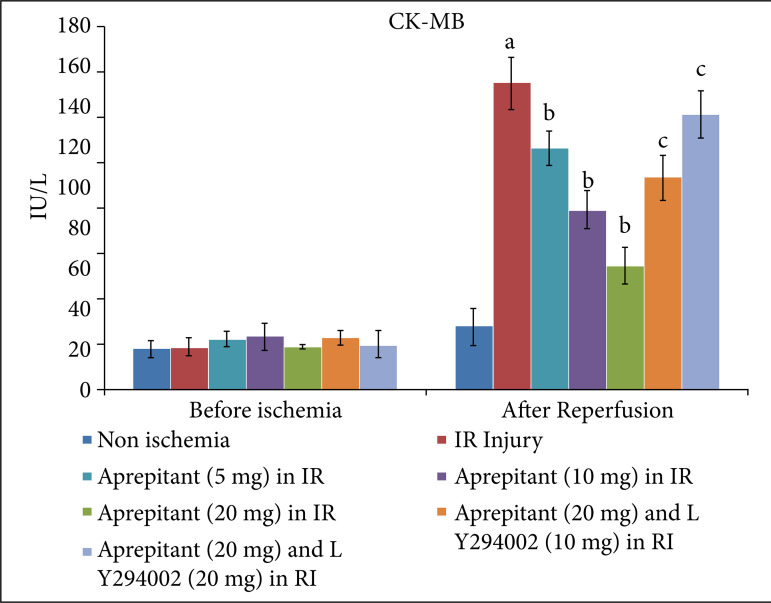
Effect of aprepitant and LY294002 on ischemia-reperfusion induced CK-MB release in the coronary effluent. Values are givenas mean ± SD. **(a)** *p* < 0.05 *vs*. non-ischemia; **(b)** *p* < 0.05 *vs*.IR injury; **(c)** *p* < 0.05 *vs*. aprepitant (20 mg/kg).

IR: ischemia-reperfusion; SD: standard deviation.

**Figure 3:**
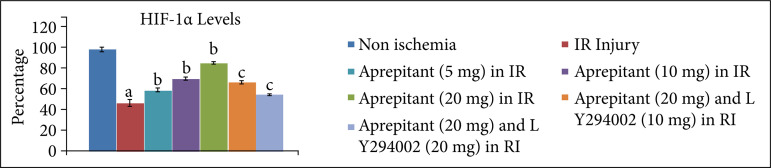
Effect of aprepitant and LY294002 on ischemia-reperfusion-induced changes in HIF-1α in the heart homogenate. Values are given as mean ± SD. **(a)** *p* < 0.05 *vs*. non-ischemia;**(b)** *p* < 0.05 *vs*. IR injury; **(c)** *p* < 0.05 *vs*. aprepitant (20 mg/kg).

IR: ischemia-reperfusion; SD: standard deviation.

**Figure 4:**
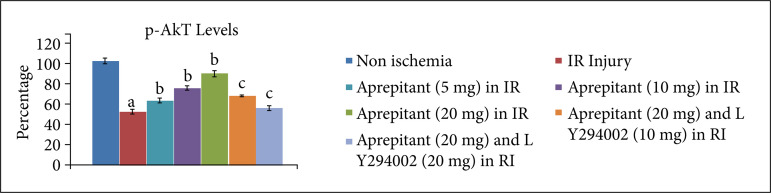
Effect of aprepitant and LY294002 on ischemia-reperfusion induced changes in p-Akt levels in the heart homogenate. Values are given as mean ± SD. **(a)** *p* < 0.05 *vs*. non-ischemia;**(b)** *p* < 0.05 *vs*. IR injury; **(c)** *p* < 0.05 *vs*. aprepitant (20 mg/kg).

IR: ischemia-reperfusion; SD: standard deviation.

**Figure 5:**
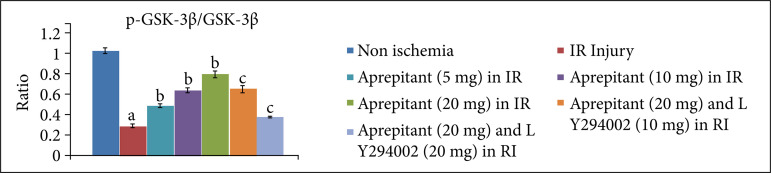
Effect of aprepitant and LY294002 on ischemia-reperfusion induced changes inp-GSK-3β/GSK-3β in the heart homogenate. Values are given as mean ± SD. **(a)** *p* < 0.05*vs*. non-ischemia; **(b)** *p* < 0.05 *vs*. IR injury; **(c)** *p* < 0.05 *vs*. aprepitant (20 mg/kg).

IR: ischemia-reperfusion; SD: standard deviation.

After giving 30 minutes of ischemia and 120 minutes of reperfusion, the levels of LDH-1 (Fig. 1, Table 1) and CK-MB (Fig. 2, Table 2) were significantly increased in the coronary effluent. The significant increase in the release of heart-injury specific biochemical indicates the presence of myocardial injury in response to ischemia and reperfusion injury. Moreover, in ischemia-reperfusion subjected rats, there was a significant change in the expression of HIF-1α, p-AkT and p-GSK-3β in the heart homogenates. There was a decrease in the levels of HIF-1α (Fig. 3, Table 3) and p-AkT (Fig. 4, Table 3) along with the decrease in the p-GSK-3β/GSK-3β ratio (Fig. 5, Table 3) as compared to non-ischemic hearts. Since p-GSK-3β represents the inactive form of GSK-3β, the decrease in the p-GSK-3β/GSK-3β ratio indicates the activation of GSK-3β enzyme.

**Table 1:** Values of LDH-1 in median (IQR) in aprepitant and LY294002 treated animals in ischemia-reperfusion induced injury.

No	Groups	Median (IQR)	
		Before ischemia	After reperfusion
1	Non-ischemic group	28.5 (4.75)	51 (12.75)
2	I/R injury	33 (7.75)	530 (37.75)
3	Aprepitant (5 mg/kg) in IR injury	27.5 (7.25)	88.5 (13.75)
4	Aprepitant (10 mg/kg) in IR injury	30.5 (6.75)	251.5 (23.25)
5	Aprepitant (20 mg/kg) in IR injury	32 (7)	456.5 (31.50)
6	Aprepitant (20 mg/kg) and LY294002 (10 mg/kg) in IR injury	27 (7.25)	250 (21.5)
7	Aprepitant (20 mg/kg) and LY294002 (20 mg/kg) in IR injury	30 (6.25)	482.5 (29.75)

LDH-1: lactate dehydrogenase 1; IR: ischemia-reperfusion; median (IQR): median with interquartile range.

**Table 2:** Values of CK-MB in median (IQR) in aprepitant and LY294002-treated animals in ischemia-reperfusion induced injury.

No	Groups	Median (IQR)	
		Before ischemia	After reperfusion
1	Non-ischemic group	19 (4.25)	29 (6.5)
2	I/R injury	20 (5.75)	155 (10.5)
3	Aprepitant (5 mg/kg) in IR injury	25 (6.0)	120.5 (9.5)
4	Aprepitant (10 mg/kg) in IR injury	24.5 (6.25)	91 (8.75)
5	Aprepitant (20 mg/kg) in IR injury	20.5 (5.75)	61 (6.25)
6	Aprepitant (20 mg/kg) and LY294002 (10 mg/kg) in IR injury	25.5 (6.25)	103.5 (9.75)
7	Aprepitant (20 mg/kg) and LY294002 (20 mg/kg) in IR injury	21.5 (5.25)	133 (10.25)

IR: ischemia-reperfusion; median (IQR): median with interquartile range.

**Table 3:** Values of HIF-?, p-AkT and p-GSK-3β/GSK-3β in median (IQR) in aprepitant andLY294002-treated animals in ischemia-reperfusion induced injury.

No	Groups	Median (IQR)		
		HIF-α	p-AkT	p-GSK-3β/GSK-3β
1	Non-ischemic group	100.5 (6.25)	102.5 (6.5)	1.05 (0.205)
2	I/R injury	46.5 (8.0)	52.5 (4.5)	0.32 (0.065)
3	Aprepitant (5 mg/kg) in IR injury	58.5 (8.5)	63 (5.75)	0.52 (0.075)
4	Aprepitant (10 mg/kg) in IR injury	69.5 (9.25)	74.5 (6.75)	0.69 (0.095)
5	Aprepitant (20 mg/kg) in IR injury	85.5 (9.75)	88.5 (7.25)	0.82 (0.085)
6	Aprepitant (20 mg/kg) and LY294002 (10 mg/kg) in IR injury	64.5 (8.25)	67.5 (6.75)	0.67 (0.080)
7	Aprepitant (20 mg/kg) and LY294002 (20 mg/kg) in IR injury	54 (6.25)	56 (5.75)	0.38 (0.06)

IR: ischemia-reperfusion; median (IQR): median with interquartile range.

### Protective effects of aprepitant on ischemia-reperfusion-induced heart injury

The administration of different doses of substance P antagonist, aprepitant (5 mg, 10 mg, 20 mg/kg), in rats 30 minutes before subjecting them to ischemia-reperfusion injury resulted in significant protection, and heart injury was significantly reduced in a dose-dependent manner. In the aprepitant-treated rats, there was a significant reduction in the release of LDH-1 (Fig. 1, Table 1) and CK-MB (Fig. 2, Table 2) in the coronary effluent. Aprepitant also restored the biochemical changes induced by ischemia and reperfusion-subjected rats. There was a significant increase in the levels of HIF-1α (Fig. 3, Table 3) and p-AkT (Fig. 4, Table 3) along with an increase in the p-GSK-3β/GSK-3β ratio (Fig. 5, Table 3). Since p-GSK-3β represents the inactive form of GSK-3β, the increase in the p-GSK-3β/GSK-3β ratio indicates the inhibition of GSK-3β enzyme.

### LY294002 abolished the protective effects of aprepitant on ischemia-reperfusion-induced heart injury

PI3K is an enzyme which leads to phosphorylation of Akt. The phosphorylated form of Akt (p-Akt) is active, and p-Akt leads to phosphorylation of GSK-3β to convert it into p-GSK-3β. Since changes were observed in the levels of p-Akt and p-GSK-3β in aprepitant-treated rats, LY294002 (PI3K inhibitor) was employed to assess its influence on aprepitant-mediated protective effects. The pre-treatment with LY294002 (10 and 20 mg/kg) significantly abolished aprepitant-mediated cardioprotective effects, and there was increase in LDH-1 (Fig. 1, Table 1) and CK-MB levels (Fig. 2, Table 1) in LY294002-pretreated rats in a dose-dependent manner. Pre-treatment with LY294002 also attenuated aprepitant-mediated restoration of HIF-1α (Fig. 3, Table 3), and p-AkT (Fig. 4, Table 3) levels along decreased in the p-GSK-3β/GSK-3β ratio (Fig. 5, Table 3).

## Discussion

In this investigation, 30 minutes of ischemia and 120 minutes of reperfusion led to rise in the levels of LDH-1 and CK-MB in the coronary effluent in comparison to non-ischemic hearts, which means the presence of significant myocardial injury. LDH-1 and CK-MB are heart-specific biochemical, and their release is used to measure the extent of myocardial injury^18^^,^ 19. Accordingly, it may be suggested that ischemia and reperfusion produced significant myocardial injury on isolated rat heart on Langendorff apparatus.

In this study, prior treatment with aprepitant (5.10, 20 mg/kg) led to significant amelioration of ischemia-reperfusion-induced increase in LDH-1 and CK-MB release in a dose-dependent manner. It suggests cardioprotective actions of aprepitant pre-treatment on ischemia-reperfusion injury. Aprepitant is neurokinin receptor blocker (NK-1 receptor) and, blocking these receptors, it attenuates the actions of substance P^6^. Aprepitant is mainly clinically used in the management of nausea and vomiting associated with chemotherapy^20^. However, there have been preclinical studies showing the usefulness of aprepitant in cardiovascular disturbances such as LDL-induced endothelial injury^21^, viral-induced myocarditis^10^ and doxorubicin-induced cardiomyopathy^11^^,^ 12. To best of our knowledge, this is the first study describing the effectiveness of aprepitant in attenuating ischemia-reperfusion-induced myocardial injury in a rat model.

In this study, the significant changes in the expression of HIF-1α, p-AkT and p-GSK-3β/GSK-3β ratio in the heart homogenates of ischemia-reperfusion injury subjected rats were also observed. There was a marked decrease in the expression of HIF-1α and p-AkT, while there was a decrease in the p-GSK-3β/GSK-3β ratio in the hearts of ischemia-reperfusion subjected rats. PI3K is an important part of the cell signalling pathway enzyme, which leads to phosphorylation and activation of Akt, and p-Akt leads to an increase in p-GSK-3β/GSK-3β ratio. GSK-3β is an enzyme whose activation is dependent on its dephosphorylation, which is opposite to many other enzymes. Therefore, an increase in p-GSK-3β/GSK-3β ratio indicates the inhibition of GSK-3β enzyme. However, aprepitant restored ischemia-reperfusion-induced decrease in the HIF-1α, p-AkT levels and p-GSK-3β/GSK-3β ratio. It suggests that the molecular mechanisms of aprepitant may involve the activation of PI3K signaling pathway involving activation of Akt and inhibition of GSK-3β. There have been previous studies showing the key role of PI3K and AkT signaling pathway in aprepitant-mediated beneficial effects^22^. To verify this contention, PI3K inhibitor, LY294002, was administered prior to treatment with aprepitant. The administration of LY294002 significantly abolished aprepitant-mediated heart protection in response to ischemia-reperfusion injury. Moreover, LY294002 also abolished aprepitant-mediated increase in the levels of HIF-1α, p-AkT and p-GSK-3β/GSK-3β ratio. The decrease in the expression of HIF-1α in response to treatment with LY294002 suggests that there is a close relationship between PI3K-AkT and HIF-1α signaling. Indeed, there have been several studies that have shown that these two signaling pathway act in concert with each other^21^^,^ 23. Furthermore, there have been studies showing that inhibition of P13K with LY294002 decreases the expression of HIF-1α^24^, which is in line with our study observation. Accordingly, it may be hypothesized that aprepitant activates PI3K enzyme to stimulate the signaling cascade involving activation of AkT and inhibition of GSK-3β signaling. Subsequently, , there may be an activation of HIF-1α signaling pathway, which may also contribute in conferring resistance to hearts against ischemia-reperfusion injury.

## Conclusion

Based on the results of the present study, it was concluded that aprepitant may be an effective drug in preventing ischemia-reperfusion-induced myocardial injury, and this protective effect may be due to activation of PI3K-AkT-GSK-3β and HIF-1α signaling pathway.
